# Evaluating the Causal Role of Gut Microbiota in Type 1 Diabetes and Its Possible Pathogenic Mechanisms

**DOI:** 10.3389/fendo.2020.00125

**Published:** 2020-03-24

**Authors:** He Zhou, Lin Sun, Siwen Zhang, Xue Zhao, Xiaokun Gang, Guixia Wang

**Affiliations:** Department of Endocrinology and Metabolism, The First Hospital of Jilin University, Changchun, China

**Keywords:** gut microbiota, dysbiosis, type 1 diabetes, causality, mechanisms

## Abstract

Type 1 diabetes (T1D) is a multifactorial autoimmune disease mediated by genetic, epigenetic, and environmental factors. In recent years, the emergence of high-throughput sequencing has allowed us to investigate the role of gut microbiota in the development of T1D. Significant changes in the composition of gut microbiome, also termed dysbiosis, have been found in subjects with clinical or preclinical T1D. However, whether the dysbiosis is a cause or an effect of the disease remains unclear. Currently, increasing evidence has supported a causal link between intestine microflora and T1D development. The current review will focus on recent research regarding the associations between intestine microbiome and T1D progression with an intention to evaluate the causality. We will also discuss the possible mechanisms by which imbalanced gut microbiota leads to the development of T1D.

## Introduction

Type 1 diabetes (T1D) is an autoimmune disease characterized by insufficient insulin production, which is caused by autoreactive T-cell-mediated partial or complete destruction of islet beta cells in patients (1, 2), with a high incidence in children and young adults (3). Genetic factors play important roles in T1D etiology, and a number of genetic loci associated with T1D have already been identified (4). Environmental factors are also a pivotal contributor to the disease (5, 6), which is evidenced in these observations that fewer than 10% of genetically predisposed individuals develop the disease (7), the increasing frequency of lower-risk genotypes in diagnosed patients (8, 9), the disparate incidence of diabetes in monozygotic twins (10), and the substantially risen prevalence in recent decades (11).

In recent years, gastrointestinal microbiota has been recognized as one of the key environmental factors associated with the development of T1D. Microbes in the human gut make up to 100 trillion cells, 10 times the number of human cells (12). More than 95% species of gut microbiota can be classified into four major microbial phyla: *Firmicutes, Bacteroidetes, Actinobacteria*, and *Protecteobacteria* (13, 14). The intestinal microbiota is sometimes described as a “hidden organ” (15) based on their capacity to perform diverse physiological functions comprising fighting against pathogens (16), producing energy (17), maintaining intestinal epithelial integrity (18, 19), and regulating immunological activities (20). There is a mutualistic relationship between the human host and the intestinal flora to maintain homeostasis (21).

A perturbation to the normal composition of commensal communities, also termed dysbiosis, may break the homeostasis and lead to various autoimmune diseases (15, 22). With the introduction of high-throughput sequencing technologies, accumulating evidence has shown that there are remarkable differences in the intestinal microbial profile between T1D patients and healthy controls, indicating a close interplay between diabetes development and gut microbiota (23). In addition, a growing number of evidence suggests that compositional changes in gut microbiota may be involved in the pathophysiology of T1D, and gut dysbiosis-mediated immunological deregulation and gut leakiness are the possible pathogenic mechanisms. However, whether these microbial changes are causal, responsive, or both so far has not been completely elucidated. Once the causal role of gut community in the onset of T1D is determined, it will provide a new opportunity for preventative or therapeutic strategies for T1D on account of the modifiable nature of gut microbiota. In this review, we will elaborate on common compositional changes of intestinal microbiome associated with T1D patients and review the evidence supporting a causal role of intestinal microbiome in the onset and progression of the disease. Furthermore, we will discuss the possible mechanisms whereby intestinal microbiome influences the T1D progression, which holds the key to unravel the complex interaction between microbiota community and host.

To determine whether there is objective evidence supporting a causal role of gut community in the onset of T1D, we reviewed literatures from different databases including MEDLINE, EMBASE, Web of Science, and the Cochrane Library. The search strategy used a combination of MeSH terms and keywords pertaining to gut microbiota and T1D. The inclusion criteria were as follows: (1) case–control or cohort studies comparing gut microbiota in patients with T1D or islet autoimmunity and healthy controls and (2) well-controlled intervention studies in human and murine models detecting the bacterial changes in fecal or mucosal samples. Studies that did not assign a control group were excluded. Two investigators independently performed the literature search and assessed the eligibility of selected studies based on the established inclusion criteria. Any discrepancies between investigators were resolved by discussion until consensus was reached. Then, we proposed the possible mechanisms whereby aberrant microbiota composition influences the T1D development.

## Aberrant GUT Microbiota Composition in Human T1D

Previous studies have found large significant differences in the microbial composition between subjects with T1D or islet autoimmunity and healthy controls (Table 1). Most of the findings originated from cross-sectional studies, which cannot directly determinize causality, but still an important initial step toward evaluating the causal role of gut microbiota in T1D pathogenesis.

**Table 1:** Gut microbiota changes in preclinical and clinical type 1 diabetes (T1D).

**Subjects studied**	**Methods**	**Changes in gut microbiota**	**References**
16 Caucasian children with T1D and 16 healthy Caucasian children	PCR-DGGE RT-qPCR	↓*Firmicutes* to *Bacteroidetes* ratio ↑*Clostridium, Bacteroides*, and *Veillonella* ↓*Lactobacillus, Bifidobacterium, Blautia coccoides*/*Eubacterium rectale* group, and *Prevotella*	(24)
12 Han Chinese subjects with T1D and 10 healthy Han Chinese subjects	16S rRNA gene sequencing	↑*Bacteroidetes*/*Firmicutes* ratio	(25)
4 children with beta-cell autoimmunity and 4 age-matched, genotype-matched, non-autoimmune individuals	16S rRNA gene sequencing	↑*Bacteroidetes* to *Firmicutes ratio* ↓Microbial diversity ↓Microbial diversity ↓Butyrate-producing species ↓*Prevotella* and *Akkermansia* ↑*Bacteroides, Veillonella*, and *Alistipes*	(26, 27)
8 Mexican children with T1D at onset, 13 children with T1D after 2 years treatment, and 8 healthy controls	16S rRNA gene sequencing	↑*Bacteroides* genus	(28)
Biopsies of the duodenal mucosa of 19 patients with T1D, 19 patients with celiac disease, and 16 healthy control subjects	16S rRNA gene sequencing	↑*Firmicutes* and *Firmicutes*/*Bacteroidetes* ratio ↓*Proteobacteria* and *Bacteroidetes*	(29)
15 children with T1D, 15 children with maturity-onset diabetes of the young 2, and 13 healthy children	16S rRNA gene sequencing	↓Microbial diversity ↑*Bacteroides, Ruminococcus, Veillonella, Blautia*, and *Strepto-coccus* genera ↓*Bifidobacterium, Roseburia, Faecalibacterium*, and *Lachnospira*	(30)
13 children at the T1D onset and 13 healthy children as control	PCR-DGGE RT-qPCR	↓Microbiota diversity ↑*Bacteroides clarus, Alistipes obesi*, and *Bifidobacterium longum* ↓*Bacteroides vulgatus, oleiciplenus, coprophilus*, and *dorei*	(31)
18 children with diabetes-associated autoantibodies, 18 autoantibody-negative children matched for age, sex, HLA-DQB1 genotype and early feeding history	16S rRNA gene sequencing	↓Lactate-producing and butyrate-producing species ↓*Bifidobacterium adolescentis* and *Bifidobacterium pseudocatenulatum* ↑*Bacteroides* genus ↓Microbial diversity	(32)
11 infants with diabetes-associated autoantibodies and 22 autoantibody-negative controls matched for gender, HLA genotype, and country	16S rRNA gene sequencing	↓Microbial diversity	(33)
28 children with new-onset T1D and 27 age-matched healthy controls	Human intestinal tract chip analysis	↑*Bacilli* (notably *streptococci*) and the phylum *Bacteroidetes* ↓Butyrate-producing species within *Clostridium* clusters IV and XIVa ↑Microbial diversity	(34)
73 children and adolescents shortly after T1D onset and 103 matched control subjects of similar place of residence and age	16S rRNA gene sequencing	↓*Clostridium* clusters IV or XIVa ↑*Escherichia* ↓*Eubacterium* and *Roseburia*	(35)
53 adults with longstanding T1D without complications or medication and 50 healthy controls matched for age, sex, and BMI	16S rRNA gene sequencing	↓Butyrate-producing species	(36)
20 patients with T1D and 28 healthy control subjects	16S rRNA gene sequencing	↑*Bacteroides vulgatus, Bacteroides rodentium, Prevotella copri*, and *Bacteroides xylanisolvens* ↓*Bifidobacterium* and *Roseburia*	(37)
Fecal protein collected from 3 T1D children and 3 control children	Combination of two-dimensional gel electrophoresis and spectral counting	↑*Clostridial* cluster XVa and cluster IV and *Bacteroides* ↓*Bifidobacteria*	(38)
35 patients with newly diagnosed T1D and 35 healthy subjects who were randomly selected and had similar demographics	Stool cultures	↓*Bifidobacterium* ↑*Candida albicans* and *Enterobacteriaceae* other than *Escherichia coli*	(39)
42 patients with newly diagnosed T1D and 42 healthy subjects	Stool cultures	↑*Candida albicans*	(40)

*PCR, polymerase chain reaction; DGGE, denaturing gradient gel electrophoresis; RT-qPCR, real-time quantitative polymerase chain reaction*.

One case–control study analyzed the gut microbiota in 16 children with T1D and 16 unaffected children. Compared with healthy control group, the *Firmicutes*/*Bacteroides* (F/B) ratio in T1D patients was significantly reduced (24). A reduction in F/B ratio was also seen in other studies. Recent studies have identified that the intestinal community profile of T1D patients in China and Turkey is also characterized by a decreased F/B ratio in comparison to healthy subjects (25, 41). Additionally, a cohort study revealed that the F/B ratio declined over time as children developed islet autoimmunity and autoimmune diabetes ultimately (26). However, conflicting data with regard to F/B ratio in individuals diagnosed with T1D have been published too (28, 29), which may be attributed to different sample sizes, data analysis approaches, and geographical location. Another common gut microbiome shift associated with the T1D development is the decreased microbial diversity, which has been reported both in T1D children (30, 31) and in autoantibody-positive children (26, 27, 32). It was further found that the decline in bacterial diversity was specific to seroconverters that eventually developed into T1D but not in undeveloped seroconverters (33). Moreover, metagenomic data from several studies showed a significant reduction in the abundance of butyrate producers such as *Clostridium* clusters IV and XIVa and mucin-degrading bacteria such as *Prevotella* and *Akkermansia* in T1D patients. A study comparing gut microbiota composition between 28 children with new-onset T1D and 27 age-matched unaffected children revealed that control children exhibited a higher number of butyrate-producing species within *Clostridium* clusters IV and XIVa compared to corresponding diabetic children (34). Children with new-onset T1D in another study also showed a similar trend regarding *Clostridium* clusters IV and XIVa (35). In addition, the decreased butyrate-producing species have been documented in adults with longstanding T1D (36) and children with pancreas autoimmunity (27, 32). In line with the reduction in butyrate-producing species that can induce mucin synthesis, Brown and colleagues have identified that *Prevotella* and *Akkermansia*, as perspective signatures of elevated mucin synthesis, were substantially lower in seropositive individuals than in healthy children (27). A decrease in *Bifidobacterium* was also reported in T1D subjects by several studies. In a study investigating the bacterial compositional differences among children with T1D and maturity-onset diabetes of the young 2 (MODY2) and healthy control subjects by 16S ribosomal RNA (rRNA) gene sequencing, the authors concluded that T1D children presented with a reduced level of *Bifidobacterium*, and perhaps even more interestingly, it was observed that the intestinal microbiota profile of T1D patients was different from not only healthy subjects but also subjects with MODY2 (30). Another study also using 16S rRNA gene sequencing concluded the similar trends regarding *Bifidobacterium* (37). Additionally, a lower proportion of *Bifidobacterium* in T1D patients was also detected by other techniques including analysis of the microbial proteome (38) and stool cultures (39). Additionally, the colonization of intestinal *Candida albicans* was also reported to be positively linked to T1D development (39, 40).

## Evidence Supporting the Causal Role of GUT Microbiota in the Pathogenesis of T1D

### Evidence From Animal Studies

In the past decade, a growing number of animal studies have suggested a causal link between intestine microflora and T1D development. This may be due to the fact that, in contrast to human studies, the gut microbiota in murine models can be disturbed under strictly controlled conditions to minimize confounding factors (42). Non-obese diabetic (NOD) mice (43) and bio-breeding diabetes-prone (BB-DP) rats (44) are two widely studied animal models of autoimmune diabetes. Both models carry the risk genes of T1D and develop T1D spontaneously (43, 45, 46).

Antibiotic intervention modulates the bacterial composition by selective removal of certain microbial lineages. It was reported that the alterations of bacterial profile in the gut induced by vancomycin remarkably increased the incidence of T1D in NOD mice (47, 48), while in another study, vancomycin displayed T1D-protecting effect in NOD mice (49). Other specific antibiotics or combinations of antibiotics have also been found to cause distinct alterations in gut microbial composition compared to untreated control NOD mice and subsequently delayed or accelerated disease progression (50–52). The types of antibiotics or the time of administration were different in each study, which may account for their disparate outcomes.

Probiotic supplement is another important intervention to change the composition of intestinal flora in NOD mice or BB-DP rats. Probiotics are defined as specific live microorganisms that can create a favorable gut environment when administered in sufficient amounts (53). Probiotics were proved to prevent diabetes development in animal models, indicating that altered intestinal microbiome has a role in manipulating the onset of T1D. A published study showed that oral administration of VSL#3, a combination of several probiotics, could prevent diabetes in NOD mice when administrated from 4 weeks of age (54). The protecting effect of VSL#3 in NOD mice was further confirmed by another study performed by Dolpady et al. (55). Probiotics *Clostridium butyricum* CGMCC0313.1 were also demonstrated to suppress the onset of T1D in NOD mice by selectively modulating the structure of gut microbiota, including increasing the F/B ratio, *Clostridium*, and butyrate-producing bacteria (56). Conferred protection against diabetes by probiotics was also shown in BB-DP rats (57).

Fecal microbiota transplantation (FMT) also can change the risk of progression to T1D by altering the gut microbiome profile in NOD mice. A study demonstrated that intestinal microflora transferred from diabetes protective NOD mice, which are genetically deficient in myeloid differentiation primary response gene 88 (MyD88), could stably alter the gut bacterial composition and eventually reduced insulitis significantly and delayed the onset of T1D in wild-type NOD mice (58). It is well-established that NOD mice harbor more diabetogenic microbes in the gut compared with non-obese diabetes-resistant (NOR) mice. A study found that the FMT from NOD mice to NOR mice elicited insulitis in NOR mice (59). Nevertheless, a recent study found that the transfer of the whole microbiota from the low- to the high-incidence colony of NOD mice did not reduce diabetes incidence. Intriguingly, single symbionts transfer of *Akkermansia muciniphila* could delay diabetes development in the high-incidence NOD colony, suggesting that individual microbiota members might have potential significance in the pathogenesis of T1D (60).

Animal studies have indicated that dietary factors such as gluten and fiber may change the incidence of T1D by altering the composition of gut microbiota. A gluten-free diet has been shown to reduce diabetes incidence in NOD mice along with elevated *Akkermansia* and reduced *Bifidobacterium, Tannerella*, and *Barnesiella* species compared to gluten-containing diet. Notably, adding gluten to the gluten-free diet reversed the antidiabetogenic effect as a result of reversing the gut bacterial composition (61). A study conducted by Toivonen et al. noted that the two fermentable fibers, pectin and xylan, exerted diabetes-promoting effect in NOD mice by significantly enhancing the level of *Bacteroides* and depleting the mucin-degrading bacteria such as *Verrucomicrobiales* and *Prevotellaceae* (62). Moreover, a recent study showed that the low esterified pectin, a novel dietary fiber, could decrease the diabetes incidence in NOD mice by selectively enriching specific microbial species that produce short-chain fatty acids (SCFAs) (63).

### Evidence From Human Studies

This chicken–egg situation (causal or consequence) can be partially clarified in several cohort studies, which have identified that the changes in gut microbiota composition occurred before the T1D development (26, 27, 32, 33). Although these prospective studies will provide a timeline of disease progression associated with microbial dysbiosis, a causal contribution can only be derived from intervention studies.

It was reported that breast milk, as an independent protective factor, could lead to a drop in the incidence of T1D (64), and the microbial composition of breast-fed infants was characterized by a more stable gut microbiome dominated by *Bifidobacteria* compared to infants who were not breastfed (65). Studies showing the protective effects of probiotics on T1D in humans are limited. One well-known study is that of Uusitalo, who previously reported that early supplementation of probiotics during the first four postnatal weeks reduced the risk of beta cell autoimmunity in infants genetically susceptible to T1D compared to those with no supplementation (66). Recently, a single-center, randomized, double-blind, placebo-controlled pilot study in children with T1D for at least 1 year showed that consumption of prebiotics could alter the gut microbiota composition and decrease the intestinal permeability, leading to improved beta cell function. However, there was no improvement in glycemic control in the prebiotic group, possibly due to the small sample size and relatively short intervention time (67). In addition, Groele et al. are conducting a double-blind, randomized, placebo-controlled study to discuss whether probiotics will improve beta-cell function by modulating the immune system in children with newly diagnosed T1D (68). Nevertheless, such randomized controlled clinical trials are still rare, and to date, there are no studies in humans supporting the link between FMT or antibiotics-induced microbial changes and T1D risk. Overall, although interventional human studies show a hint of promise, a causal relationship still cannot be concluded due to the lack of large-scale prospective studies demonstrating that long-term changes in the bacterial composition alter T1D risk. Further randomized controlled studies in large human cohorts will need to be undertaken.

Collectively, the evidence generated from well-controlled intervention studies in murine models is promising for establishing a causal relationship. However, conclusions from animal studies must be interpreted with caution, as animal models have a number of limitations, making it difficult to translate to humans. In addition, human studies showing a clear association between the long-term changes of gut microbiota and altered T1D risk are still lacking. Thus, the evidence supporting causative relationship summarized above is inconclusive and needs to be further confirmed.

## The Possible Mechanisms Whereby GUT Microbiota Influences the T1D Development

### Immunological Deregulation and Leaky Gut Are Involved in T1D Development

**Figure 1:**
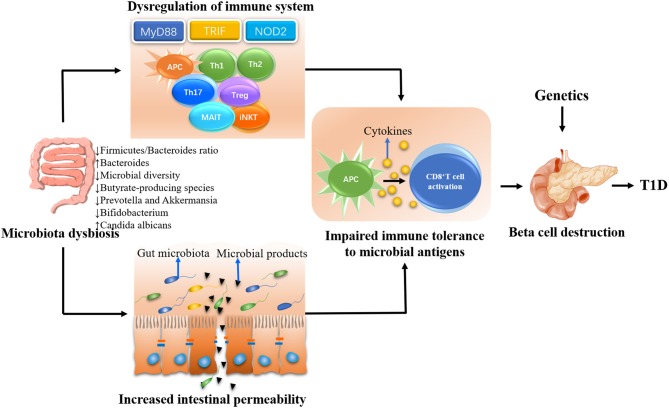
The possible mechanisms whereby gut microbiota influences the type 1 diabetes (T1D) development. The gut microbiota plays a decisive role in the maturation of immune system in early life. Gut dysbiosis will lead to the dysregulation of immune response including both innate and adaptive immune system, eventually resulting in beta cell destruction and the onset of T1D in genetically susceptible individuals. On the other hand, the gut dysbiosis can lead to the disassembly of tight junctions, thereby disrupting the integrity of intestinal barrier. The enhanced intestinal permeability will allow unregulated passage of microbial antigens such as microbiota and their products. These antigens escaping from intestinal tract could be untaken by antigen-presenting cells (APCs), which can process and present antigens to autoreactive T cells and subsequently promote the destruction of pancreatic beta cells in genetically predisposed individuals.

There was a proposed complicated interaction between intestinal microbiota, immune system, and gut permeability (69). A large body of evidence suggested that defective profile of intestinal microbiome may influence the pathogenesis of T1D by affecting immune homeostasis and/or gut permeability (Figure 1) (70).

Gastrointestinal microbiota has been considered as the largest organ of the immune system (71) owing to their ability to constantly interact with immunological cells (20). It was demonstrated that there are disorders of the mucosal immune system in NOD mice before diabetes onset (72). Gastrointestinal microflora plays an essential role in the development and maturation of the immune system, and early stages of life are the critical time window for the establishment of immune tolerance (13, 73). According to the Hygiene Hypothesis, it is the lack of microbial stimulation in early childhood resulting from advances in medicine and improved sanitation that leads to the rise in the prevalence of immune-related disorders (74). Thus, the infants, who can benefit from early exposure to specific microorganisms, may be much less likely to develop autoimmune diseases later in life (75, 76). The Hygiene Hypothesis has been supported by murine studies. For example, NOD mice were more likely to develop diabetes when they lived in a clean environment, and it was reported that infection of NOD mice with various bacteria in early life could prevent T1D development (77, 78). In general, hence, it seems that gut microbiome-mediated appropriate immune maturation during early life is critical to prevent T1D development (79). Conversely, alterations of gut microbiota composition will lead to a poorly educated immunity and eventually result in insulin-secreting beta cell damage and the onset of T1D in genetically predisposed subjects (80, 81).

Another proposed T1D pathogenesis associated with microbial dysbiosis is the increased intestinal permeability, often referred to as a “leaky gut” (69), which may act either independently or coincidently with the immunological deregulation (82). A “leaky gut” in T1D has been observed from both human studies and animal research (83–86), indicating that the barrier dysfunction is a primary feature of T1D. Some researchers argued that hyperglycemia and insulitis may contribute to the enhanced leakiness of the gut epithelial barrier in T1D (87). Nevertheless, other researchers favor the notion that the “leaky gut” may be more of a cause than an effect, as a highly permeable gut has been observed before the development of both insulitis and clinical onset of T1D (88, 89). One of the most cited studies is that of Bosi et al., who measured intestinal permeability of 81 subjects at different stages of T1D as well as 40 healthy controls. They found that the increased gut permeability occurred before the manifestation of the disease (90). Similarly, a recent study performed by Harbison and others observed that children at risk for T1D had higher intestinal permeability and were associated with gut microbiota dysbiosis (91). Furthermore, administration of hydrolyzed casein diet has been reported to be able to protect from T1D by improving the gut integrity in BB-DP rats (92), indicating that the melioration of increased gut permeability can protect against autoimmune diabetes development. In line with this notion, infection with barrier-disrupting *C. rodentium* accelerated insulitis in NOD mice, while NOD mice failed to develop insulitis when infected with the *DeltaespF* strain, which cannot disrupt gut integrity (93). These studies support the concept that such a “leaky gut” could be a component within the natural history of T1D progression and perhaps is the initial steps of evolution to the disease. It was suggested that the enhanced intestinal permeability, as a consequence of impaired integrity of the intestinal barrier (94), might allow unregulated passage of exogenous antigens especially the microbial components (95). The translocation of these microbial components to systemic compartment could trigger systemic inflammation and autoimmune progression by directly damaging pancreatic beta cells (96). Alternately, these microbial components could be untaken by antigen-presenting cells (APCs), which can process and present the antigen to autoreactive T cells (87, 97), leading to the destruction of islet beta cells (57, 89). Another possible mechanism by which these translocated microbial antigens initiate the diabetes onset is molecular mimicry. Some microbial antigens have homology with islet self-antigen and may result in the destruction of islet beta cells by T-cell cross-reactivity (98). A recent study reported that oral administration of *Bacteroides fragilis* under loss of gut barrier integrity condition induced by chemical approach could lead to rapid disease progression in NOD mice, further highlighting the role of microbial translocation in contributing to T1D (99).

Understanding such mechanisms through which gut microbiota influences the T1D development is of great importance for developing novel prevention and treatment strategies of autoimmune diabetes. However, before making much research effort on microbiota-based therapies, it would seem to be pivotal to uncover first whether and how dysbiotic gut microbiota contributes to immunological aberrancies and gut leakiness.

### Impact of Gut Microbiota on the Immune System

Alterations of the intestinal microbiota during early life have been hypothesized to impact T1D pathogenesis by disturbing the normal pattern of immunological maturation (81), and various bacterial taxa and bacterial metabolites as well as bacteria-derived components, which were able to affect immune responses, have been identified (100, 101).

#### Impact of Gut Microbiota on the Innate Immune System

Innate immunity plays an essential role in the etiology of T1D, and investigations showed that interaction of commensal bacteria with the innate immune system was involved in the onset and progression of T1D (3). Toll-like receptors (TLRs), known as important players in the innate immunity, are critical for intestinal homeostasis. TLRs as one of pathogen recognition receptors (PRRs) expressed on immune and non-immune cells can recognize pathogen-associated molecular patterns (PAMPs) derived from microbiota (102) and enable the initiation of the innate immune system (103). There are many different microbes that can facilitate or inhibit autoimmunity of T1D by signaling through different receptors of the TLR family (104–108). The first attempt to investigate innate immune pathway associated with microbial exposure in T1D was conducted in MyD88-deficient NOD mice. MyD88 is an adaptor protein of multiple TLRs that can recognize microbial stimuli and contribute to downstream signaling pathways of TLRs (102, 109). NOD mice deficient in molecule MyD88 were completely protected from T1D under conventional conditions, and the protective effect was derived from beneficial microbial composition, which differed from that of wild-type controls, indicating that the composition of microbiota was changed by host MyD88 deficiency (3). Conversely, MyD88-deficient mice had an increased risk of developing T1D under germ-free (GF) conditions, while the incidence of diabetes was reduced in these mice when exposed to a defined microbial mixture, which further supports the intimate interaction between microbial community and host innate immune system (3). A recent study conducted by Gulden and colleagues unraveled a novel innate immune pathway influenced by gastrointestinal microbiota in T1D development. They found that the deletion of the Toll/interleukin-1 receptor (TIR)-domain-containing adapter-inducing interferon-β (TRIF), another critical adaptor protein downstream of TLRs, could protect NOD mice from diabetes. Importantly, a different microbiota profile was found in TRIF-deficient NOD mice compared to wild-type NOD mice, suggesting that the protective effect of TRIF deficiency is through changing microbial composition (110). In addition, a recent study performed by Simon et al. found that NOD TLR4^−/−^ animals had an increased risk of progression to diabetes along with higher abundance of *Bacteroidetes* and lower *Firmicutes* in the large intestine before the onset of T1D when compared to NOD TLR4^+/+^ mice, indicating that TLR4 expression status determined early alterations of gut microbial composition (111). NOD-like receptors (NLRs), another important PRR, have also been reported to be involved in T1D development through the recognition of bacterial products. Costa et al. found that NOD2 receptor could be activated by translocated gut bacteria in pancreatic lymph nodes (PLNs) of streptozotocin (STZ)-treated mice and contribute to the pathogenesis of T1D, while broad-spectrum antibiotics treatment and NOD2 receptor deletion could protect STZ-treated mice from T1D (112). Additionally, it has been documented that early-life gut microbiota is critical to the development and function of APCs (113). Studies conducted by Hu et al. found that antibiotic-treated NOD mice displayed protective effect for T1D primarily achieved by the generation of immune-tolerogenic APCs, which possessed the impaired self-antigen presentation function and eventually led to reduced activation of cytotoxic CD8^+^ T cells. Interestingly, the tolerogenic APC-mediated diabetes protection was able to be transferred to the second generation (48, 50). This mechanism of tolerogenic APCs induced by changes in the intestinal microbiome is reinforced by a study investigating macrophages, a potent subset of APCs, in which these cells showed hyporesponsiveness to lipopolysaccharide (LPS) stimulation in streptomycin and cefotaxime-treated mice, whose gut microorganisms were nearly completely eliminated after 3 weeks treatment (114). Dendritic cells (DCs), another major subset of APCs, have also been demonstrated to become tolerogenic when exposed to specific microbial stimuli including *Lactobacillus reuteri* (115) and a mixture of *Bifidobacteriaceae, Lactobacil-laceae*, and *Streptococcus* (55).

#### Impact of Gut Microbiota on the Adaptive Immune System

Intestinal immune health is achieved, in part, through the development of adaptive immune response (116), while perturbations in the composition of gut microbiome can affect the adaptive immune system development at multiple levels including CD4^+^ T cells, CD8^+^ T cells, mucosal-associated invariant T (MAIT) cells, and invariant natural killer T (iNKT) cells.

T helper 1 (Th1) and T helper 2 (Th2) cells as major components of the adaptive immune response are vital for controlling the autoimmune reactions (117). An imbalanced Th1/Th2 response has been reported to be involved in the damage of islet beta cells in T1D (117). It has been demonstrated that the commensal community has a decisive role in establishing this equilibrium (118, 119). For instance, GF mice present with a highly Th2-skewed cytokine profile (120, 121), and a shift from Th2 to Th1 immune response has been identified to be promoted by exposures to microorganisms early in life (118). The maturation of regulatory T cells (Tregs) expressing Foxp3 transcription factor is crucial for immune homeostasis and induction of tolerance (122). Numerous studies have revealed that reduced frequency or function of Foxp3^+^ Tregs in NOD mice is a major susceptibility factor for T1D (123–125). Notably, the Foxp3^+^ Tregs in mesenteric lymph nodes of GF mice showed a significant reduction in relative and total numbers as well as an impaired regulatory function, indicating a critical role of the gut microbiota in regulating Tregs development (126). Interestingly, monocolonization of GF mice with *B. fragilis*, a member of the *Bacteroidetes* phyla, could restore the differentiation of Foxp3^+^ Tregs (121, 127). Other defined bacterial strains that have the capacity to increase Foxp3^+^ Tregs number and function in GF mice are *Roseburia faecis* (a member of *Clostridium* cluster XIVa) and *Faecalibacterium prausnitzii* (a member of *Clostridium* cluster IV) (128). It has been postulated that these microbes induce Tregs by producing butyrate (129). Intestinal commensal microorganisms can ferment the dietary fibers and produce SCFAs such as acetate, propionate, and butyrate (130). Butyrate can not only provide energy for colonic epithelial cells (131) but also enhance the abundance and function of splenic and colonic Foxp3^+^ Tregs via histone modification (15, 132). Th17 cells have also been considered crucially involved in the etiology of T1D (133, 134). The main function of Th17 cells is producing interleukin (IL)-17 and clearing the extracellular pathogens during infection, while an excessive inflammatory response triggered by Th17 cells may promote autoimmunity (135). However, much uncertainty still exists about its role in autoimmune diabetes because a diabetes-protective effect of Th17 immunity has also been reported in T1D (136). Alam et al. found that the level of IL-17 expression was decreased in the small intestinal lamina propria of GF mice (137). Of note, the reduced frequencies of Th17 cells in GF mice could be restored upon colonization with segmented filamentous bacteria (SFB) (138). At the same time, it was demonstrated that SFB colonization could protect male GF NOD mice from diabetes development and conferred a protective effect in female NOD mice when there were other intestinal microbes (139). Moreover, there are other Th17 cells inducers such as altered Schaedler flora (ASF). ASF, a mixture of eight intestinal bacterial species, also has the capacity of inducing the Th17 responses in GF mice (140). CD8^+^ T cells are essential for beta cell destruction (141). A recent study revealed that insulin-reactive pathogenic CD8^+^ T cells were activated as a result of bacterial composition change caused by antibiotic treatment and thus led to the acceleration of diabetes progression in a NOD transgenic mouse model (142), suggesting that compositional change are likely responsible for CD8^+^ T cells regulation. Recently, Rouxel et al. suggested that MAIT cells known as innate-like lymphocytes might exert protective impact on T1D by maintaining gut integrity and controlling anti-islet autoimmune responses (143). It has been reported that the number of MAIT cells in GF mice was lower than that of specific pathogen-free mice, indicating a close interplay between MAIT cells and gut commensals (144). The iNKT cells have also been recently considered to play a key pathogenic role in the development of T1D since the cytokine-secretion phenotype of iNKT17 cells, mainly IL-17-producing phenotype, could directly trigger autoimmune diabetes (145). It was identified that the gastrointestinal microbiota could promote iNKT17 cell differentiation and acquire a specific cytokine-secretion phenotype (146). One piece of evidence supporting this notion is that GF mice displayed less mature and hyporesponsive iNKT cells (147, 148). In addition, De Giorgi et al. found that the increase in iNKT17 cell differentiation in NOD mice correlated with specific intestine bacterial composition, which was characterized by increased microbial richness, elevated frequency of *Bacteroidales*, and reduced relative abundance of *Clostridiales* strains (149).

### Impact of Gut Microbiota on Intestinal Permeability

There is emerging evidence suggesting that the permeability of the intestinal barrier can be regulated by microbial community (72, 150–153). Several commensals have been demonstrated to have a beneficial impact on mucosal barrier integrity, while some other bacteria exert adverse effects (154).

In recent years, a considerable amount of studies have recognized a negative correlation between butyrate-producing species and the risk of T1D, as already mentioned above (27, 32, 34, 36). These butyrate-producing species have been reported to have a pivotal role in the maintenance of intestinal barrier integrity. It was identified that butyrate could reduce gut permeability by promoting the assembly of tight junctions (TJ) (155) as well as inducing mucin synthesis (131), and the role of butyrate in restoring TJ barrier was achieved by affecting the expression of TJ proteins comprising claudin-2, occludin, cingulin, and zonula occludens (ZO) proteins (156). Despite the great variability of intestinal microbiota associated with T1D, most published studies have found that *Bacteroides* were positively associated with T1D development (24, 27, 28, 30, 32, 34, 38). These bacteria are able to ferment glucose and lactate to propionate, acetate, and succinate (157), which cannot induce the biosynthesis of mucin-like butyrate (158). Conversely, these bacteria would reduce the assembly of TJ and generate an increase in gut permeability, eventually promoting the T1D-associated autoimmunity (27, 150). Collectively, the bacteria capable of converting lactate to butyrate contribute to increased mucin synthesis and TJ formation, thereby facilitating gut health. In contrast, bacteria metabolizing lactate to other SCFAs are related to impaired TJ with a consequential increase in gut permeability and result in the onset of T1D (27). Certain producers of lactate may produce net butyrate (159); thus, these bacteria may also be of great significance for the intestinal barrier function. For instance, *Bifidobacterium longum* subspecies *infantis*, lactate-producing bacteria, were demonstrated to have the capacity to protect intestinal permeability (160). Several bacteria with probiotic effect including *Lactobacillus johnsonii* N6.2 (57), *B. lactis* (161)*, Lactobacillus rhamnosus* and *Lactobacillus reuter* (162), and *Lactobacillus plantarum* (163) were also reported to be able to decrease intestinal permeability (164).

However, as yet, the mechanisms underlying the modulation of epithelial barrier function by gut microbiota are complex and remain unclear (152). One of the potential pathways through which gut microbiota affects intestinal permeability appears to be dependent on high levels of zonulin, whose production can be regulated by bacterial colonization (165, 166). Zonulin has been discovered to reversibly regulate intestinal permeability by modulating TJ (95, 167, 168). It was reported that there was increased zonulin release coincident with an increased permeability before the onset of clinically evident T1D (5). It was supposed that imbalanced microflora colonization could induce the upregulation of zonulin into gut lumen (7). The released zonulin was then recognized by receptors on the surface of intestinal epithelial cells and elicited changes in TJ dynamics including the remodeling of cytoskeleton and the phosphorylation of ZO-1 and occludin (169). In the end, gut permeability was enhanced as a consequence of the disassembly of TJ (169, 170).

## Conclusions

A large number of studies have demonstrated that the altered abundance of specific members or reduced diversity of gut microbiota was associated with the progression of T1D. However, the exact role of gut microbiota in the pathogenesis of T1D remains controversial. Up to now, the most convincing evidence for a causal link between intestinal microbiome and the disease comes from well-controlled intervention studies in murine models. These studies illustrated the efficacy of probiotic supplement, antibiotic use, FMT, and diet intervention in modifying the risk of T1D via changing the gut colonization patterns. Furthermore, increasing evidence has indicated that the involvement of intestinal microflora in T1D pathogenesis may be through exerting impact on immune homeostasis and/or gut permeability. Taken together, these findings reviewed here underscore the importance of maintaining healthy microbial composition and provide a new insight into T1D prevention or treatment measures. However, translating these insights into feasible therapeutic measures might be challenging. Further randomized controlled studies in large human cohorts are needed to help answer this chicken–egg question and confirm the efficacy of microbiota-based therapeutic approaches.

## Author Contributions

HZ designed this review. LS, SZ, and XZ performed the paper selection. HZ, GW, and XG wrote the manuscript. All authors approved it for publication.

### Conflict of Interest

The authors declare that the research was conducted in the absence of any commercial or financial relationships that could be construed as a potential conflict of interest.
